# 731 Utilizing call for burn admissions to guide clinical staffing decisions for Advanced Practice Providers (APP)

**DOI:** 10.1093/jbcr/irac012.285

**Published:** 2022-03-23

**Authors:** Atique Alam, Beretta C Coffman, Yusuf Presberry, Joan Wilson

**Affiliations:** Burn & Reconstructive Centers of America, Augusta, Georgia; Joseph M. Still Burn Center at Doctors Hospital, Augusta, Georgia; Joseph M. Still Research Foundation, Inc, Augusta, Georgia; Joseph M. Still Burn Center at Doctors Hospital, Augusta, Georgia

## Abstract

**Introduction:**

Over the past decade, the Advanced Practice Provider (APP) role has expanded into various care settings. The literature confirms that APPs contribute positively to the ICU setting, where better patient outcomes are noted, and they are seen as a safe adjunct to the ICU team. The VA recognizes the need for a more significant presence, and practice-based variations are considered crucial as restructuring goals are established. In 2014, Edkins et al. recognized the need for APPs in the burn setting as the number of residents declined. They stressed appropriate utilization and ongoing educational opportunities to develop the role in burns further.

**Methods:**

This project was conducted by establishing a protocol within our burn referral call system to adequately and efficiently ensure accurate data is accumulated and leveraged to make staffing decisions. Implementation of the protocol began in January of 2018 and is well established. The primary purpose of the protocol is to efficiently store and transfer patient data to make informed decisions across both clinical and administrative departments. The Customer Relationship Management (CRM) System, Salesforce, was the primary software to implement and maintain this process.

**Results:**

For three years, a total of 42,460 referral calls came through our burn call system. The number of patient calls that required admission to a regional burn center totaled 15,293. 70% of total patient referral calls came during the hours of Noon-Midnight; similarly, 72% of admission patient calls came during Noon-Midnight. Hourly trends were similar for both total patient call volume and admission patient referral call volume as they both had the highest number of referral calls between the hours of Noon-Midnight. The majority (32%) of total patient referral calls came in during earlier days of the week (Monday and Tuesday), while the majority (30%) of admission patient referral calls came in during the later days of the week (Friday or Saturday).

**Conclusions:**

Our study demonstrates the benefits of implementing a collaborative protocol that allows sharing data and information across all facets of a burn center. Opportunities for additional studies include implementing similar protocols for administrative staffing and analysis of patient outcomes to see how staffing utilization has affected patient care.

